# Spectrum of diabetes mellitus in patients with Shwachman-Diamond syndrome: case report and review of the literature

**DOI:** 10.1186/s13052-023-01501-z

**Published:** 2023-08-14

**Authors:** Lusine V. Navasardyan, Ingrid Furlan, Stephanie Brandt, Ansgar Schulz, Martin Wabitsch, Christian Denzer

**Affiliations:** 1 Department of Endocrinology, Arabkir Medical Center, Yerevan State Medical University, Yerevan, Armenia; 2 Department of Pediatrics and Adolescent Medicine, University Medical Center Ulm, Ulm, Germany; 3 Division of Pediatric Endocrinology and Diabetes, Department of Pediatrics and Adolescent Medicine, University Medical Center Ulm, Eythstr. 24, 89075 Ulm, Germany

**Keywords:** Shwachman-Diamond syndrome, Pancreatic exocrine insufficiency, Diabetes mellitus, Case report

## Abstract

**Background:**

Shwachman-Diamond syndrome (SDS) is a rare congenital disorder caused by mutations in the SBDS gene and characterized by exocrine pancreatic deficiency, hematologic dysfunction, and skeletal growth failure. Although the hematologic features and characteristics of the somatic disorders commonly associated with SDS are well known, emerging data from case reports and patient registries suggest that SDS may also be associated with an increased risk of diabetes mellitus. However, currently available data on SDS-associated diabetes are limited and do not allow conclusions regarding prevalence and incidence rates, clinical course, and outcomes.

**Case presentation:**

Here we report the case of a 5-year-old girl with SDS who underwent bone marrow transplantation at the age of 3 months and developed autoantibody-positive type 1 diabetes mellitus at the age of 1.8 years. The manifestation and course of diabetes development were mild, complicated by concurrent spontaneous episodes of hypoglycemia even before the onset of antidiabetic treatment. Currently, adequate metabolic control can be achieved by dietary intervention.

**Conclusions:**

Considering that the SBDS protein regulates mitosis and ribosomal biosynthesis and that its suppression may cause immunologic instability and chronic inflammation, this case provides insight into the phenotype of rare Shwachman-Diamond syndrome-associated diabetes mellitus, which may be characterized by significant age-dependent differences in clinical course.

## Background

Shwachman-Diamond syndrome (SDS) is a rare congenital multisystem disorder characterized by exocrine pancreatic insufficiency and bone marrow dysfunction with an increased risk of leukemia or bone marrow aplasia, growth failure, and short stature [1].

The birth prevalence is reported to be 1/153,000–168,000 live births, mostly of autosomal recessive inheritance, with a male/female ratio of 1.7/1. No specific ethnic predilection has been identified [2].

The genetic causes of SDS in 80–90% of cases are mutations in the SBDS gene (7q11.22), which encodes a protein responsible for ribosome biogenesis and mitosis and is expressed in all human tissues [3, 4]. In some cases, SDS can also be caused by DNAJC21, EFL1, or SRP54 gene mutations [5–8]. Of note, SRP54 gene mutations are inherited in an autosomal dominant pattern [9, 10].

Clinical symptoms manifest during infancy and early childhood and show wide variability. The most common feature of SDS is moderate, intermittent neutropenia associated with recurrent secondary infections. Mild anemia and thrombocytopenia may also be seen. Exocrine pancreatic insufficiency is characterized by steatorrhea, failure to thrive, and growth retardation. Despite adequate nutrition and pancreatic enzyme replacement, 38% of children with SDS are reported to have height and weight below the third percentile for age and sex [11]. Delayed bone age and maturation with metaphyseal dysplasia result in short stature, pectus carinatum, osteopenia, and abnormal somatic development [12, 13]. Other clinical features may also occur, including dental abnormalities, psychomotor retardation, eczema or ichthyosis, and mental retardation (Table 1).

**Table 1:** Clinical features of the reported SDS patient with diabetes mellitus categorized as either typical or nontypical for Shwachman-Diamond syndrome

Summary of clinical and laboratory findings in the reported patient		
	**typical for SDS**	**nontypical for SDS**
**Hematologic findings**		
Thrombocytopenia, anemia and agranulocytosis in the newborn period		✓
Bone marrow failure requiring stem cell transplantation at the age of 3 months		✓
Neutropenia	✓	
Recurrent secondary infections: sepsis, labial abscess, bronchitis, gastroenteritis etc	✓	
**Nutrition, exocrine and endocrine pancreatic function**		
Congenital pancreatic exocrine insufficiency	✓	
Recurrent spontaneous hypoglycemia		✓
Diabetes mellitus type 1		✓
Severe eating disorder, no oral food intake at age 5 ys		✓
**Growth and development**		
Pulmonary hypertension		✓
Muscular hypotonia	✓	
Growth retardation, short stature, failure to thrive	✓	
Psychomotor retardation, intellectual disability	✓	

The overall prognosis of SDS depends on the clinical severity of the manifestations and in particular on the development of bone marrow failure. Approximately 1/3 of patients develop bone marrow aplasia and subsequent leukemic transformation requiring bone marrow transplantation. However, the risk of malignant transformation from bone marrow failure to myelodysplastic syndrome in SDS patients is not well defined, although the risk of leukemic transformation seems to increase with age [14].

### Diabetes mellitus in SDS patients

Type 1 diabetes mellitus is caused by autoimmune destruction of pancreatic beta cells, resulting in absolute insulin insufficiency. To date, diabetes mellitus and pancreatic endocrine dysfunction have not been associated with SDS. In a case series of 25 genetically proven SDS patients, 26% were affected by at least one endocrine disorder (excluding short stature). Interestingly, there was only one case of manifest type 1 diabetes mellitus (4%), but 5 patients were found to have either impaired fasting glucose or impaired glucose tolerance (fasting glucose > 100 mg/dl or postprandial glucose > 140 mg/dl in a standard oral glucose tolerance test) [11]. In the Italian national SDS registry, type 1 diabetes was reported in 3.23% (2 of 62) of cases, indicating a significantly increased risk of diabetes in SDS patients compared to the reference population [15]. The authors hypothesize that known defects in immune regulation in SDS may play a role in the pathogenesis of type 1 diabetes mellitus. Furthermore, defective ribosome function in SDS may contribute to impaired insulin secretion and predispose affected patients to the development of dysglycemia [16].

To our knowledge, a total of 11 cases of type 1 diabetes in SDS patients have been reported [13, 15, 17–24]. It should be noted that only 4 of these 11 patients were reported to have type 1 diabetes with positive autoantibody screening, while the remaining patients had either negative or no measured antibodies. Furthermore, no common pathogenetic mechanism has been identified to account for the potential increased risk of diabetes development in SDS patients.

From an epidemiologic point of view, there is very limited information on possible age peaks at onset. Among the published cases, the youngest was a premature infant with SDS who developed autoantibody-negative diabetes mellitus at the age of three months due to slowly progressive hyperglycemia requiring insulin treatment 12 months later [20]. Another case of neonatal manifestation of diabetes in a patient with SDS was treated with tolbutamide with efficient glucose normalization over the years [17]. Kamoda and coworkers described the case of an infant with SDS with slowly deteriorating glucose homeostasis from 3 weeks of age until the diagnostic criteria for overt diabetes mellitus were met at 15 months, requiring insulin therapy. In this case, hyperglycemia remained mild for the first 12 months, suggesting a slow and gradual decline in beta-cell capacity [21].

There is also a paucity of data on the treatment options used, the metabolic control achieved, and the acute and chronic complications of diabetes mellitus in Shwachman-Diamond patients. One case report showed improved health-related quality of life after initiation of continuous subcutaneous insulin infusion (CSII) in a boy with SDS who developed antibody-negative diabetes at the age of 13 years [24]. Recently, another child with type 1 diabetes and SDS was also reported to be effectively treated with insulin pump therapy (CSII) [13].

Interestingly, severe episodes of symptomatic hypoglycemia in the context of otherwise normal glucose regulation have been reported in a neonatal patient with SDS requiring treatment with diazoxide and in another child with SDS at the age of 2 years [25, 26]. According to these case reports, the underlying pathogenesis of recurrent hypoglycemia has not been identified, and the hypoglycemia appears to resolve with age.

Reviewing all reported cases of diabetes mellitus in SDS patients, we hypothesize that SDS-associated diabetes can be divided into two groups: first, cases with a rather mild course of diabetes after manifestation in infancy or early childhood [17, 20, 21, 26], and second, cases in older children or adolescents that rapidly require intensive insulin therapy as in classic type 1 diabetes [13, 24].

### Other endocrine comorbidities in SDS patients

No consistent endocrine phenotype has been observed in patients with SDS with a genetically confirmed diagnosis [11, 13]. Endocrine disorders other than diabetes mellitus that have been described in patients with SDS include growth hormone deficiency, hypothyroidism, congenital hypopituitarism, and post-transplant hypergonadotropic hypogonadism [11]. Systematic data on the incidence of endocrine abnormalities in patients with SDS are not currently available.

## Case presentation

### Introduction and patient information

We report here the case of a 5-year-old girl who underwent bone marrow transplantation for SDS-associated bone marrow failure at a very young age, presenting with a slowly progressive development of dysglycemia characterized by spontaneous hypo- and hyperglycemia.

The child was born into a family without any specific previous history; only a paternal cousin had mental retardation of unknown origin. The girl was born at 37 + 6 weeks' gestation with a weight of 1920 g (500 g < 3rd percentile) and a length of 43.5 cm (2.5 cm < 3rd percentile), indicating small for gestational age (SGA). She was born with a connatal infection (an indication for antibiotic therapy) and was placed on CPAP therapy for respiratory adaptation disorder, which was discontinued on day 4 of life. At birth, she had a postnatal petechial rash on the extremities and was found to have severe thrombocytopenia, which was treated with platelet concentrate substitution. Due to severe muscular hypotonia, nutrition was initially provided only through a gastric tube. Genomic DNA sequencing was performed on the basis of clinical features with marked muscular hypotonia, laboratory findings (tricytopenia: thrombocytopenia, microcytic hypochromic anemia, agranulocytosis) and apparent pancreatic insufficiency (significantly decreased pancreatic enzymes in serum and stool) suggestive of Shwachman-Diamond syndrome (Table 1). SDS was diagnosed based on the molecular genetic detection of two compound heterozygous mutations in the SBDS gene: SBDS, exon 2, c.183_184delTAinsCT, pLys62delins*, and SBDS, exon 4, c.523 > T, p.Arg175Trp. The detected mutations are inherited in an autosomal recessive manner: the 183_184TAinsCT mutation is reported to be one of the most common causes of SDS [26–28], and the c.523 > T mutation was first described in 2006 in a 5-year-old patient with a severe, distinctive clinical picture of SDS [29].

### Clinical findings and timeline

At the age of 26 days, the patient was transferred to the Immunology and Stem Cell Transplantation Clinic. During her continuous hospitalization for the first six months after birth, she suffered from an abscess of the right labia with a recto-cutaneous fistula (released by surgical intervention) and recurrent secondary infections requiring antibiotic therapy. She received regular platelet and erythrocyte replacement and was started on pancreatic enzyme replacement therapy. She continued to require tube feeding and supplemental parenteral nutrition, but did not achieve adequate weight gain. At 3 months of age, she underwent allogeneic bone marrow transplantation from her HLA-haploidentical father (Table 1).

Treatment with glucocorticoids was started 2 weeks after bone marrow transplantation for obliterative bronchiolitis requiring oxygen supplementation. She remained in the hospital until 10 months of age and was readmitted at 1 year of age for pulmonary hypertension and partial respiratory failure with persistent oxygen requirements. Pulse therapy with methylprednisolone was started at the age of 1 year 3 months because of suspected pulmonary GvHD. It is noteworthy that the patient passed this period without hyperglycemia. Thereafter, treatment with hydrocortisone was continued until the age of 2 years and 8 months. From the age of 1 year, recurrent hypoglycemic episodes were observed, with spontaneous glucose levels as low as 39 mg/dl, notably without ever presenting clinical symptoms of severe hypoglycemia.

### Diagnostic assessment

Diagnostic workup of spontaneous hypoglycemia was attempted twice under inpatient conditions, but was terminated prematurely and without diagnostic results each time at the request of the parents. Repeated changes in tube feeding formulas and modifications of feeding times and duration did not improve the incidence and frequency of hypoglycemic episodes at this time.

At 2.8 years of age, she was started on regular immunoglobulin replacement for chronic IgG deficiency. At the age of 4 years, neurological examination revealed that cognition, motor development, and speech development status corresponded to developmental standards of 10–27 months. Throughout the course, the girl did not eat or drink per os, so nutrition and hydration were entirely dependent on gastric tube feeding, combined with additional parenteral nutrition via a central venous line at night until the age of 3 years. Table 1 provides a comprehensive overview of the clinical phenotype of our patient and categorizes the patient's characteristics into typical and atypical conditions in SDS patients.

### Course of diabetes manifestation

**Fig. 1:**
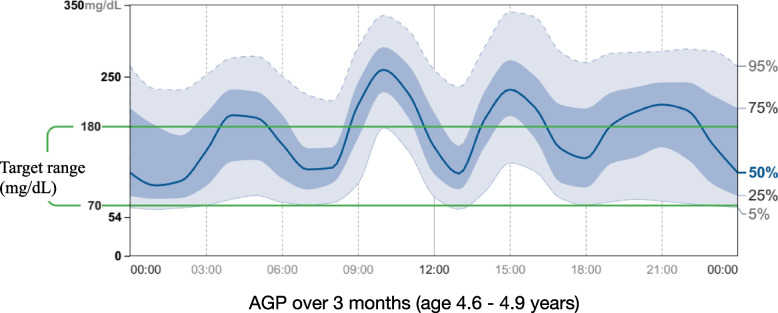
Ambulatory glucose profile (AGP) over 3 months of our patient with Schwachman-Diamond syndrome (age 4.6 to 4.9 years) before the most recent diagnostic work-up

**Fig. 2:**
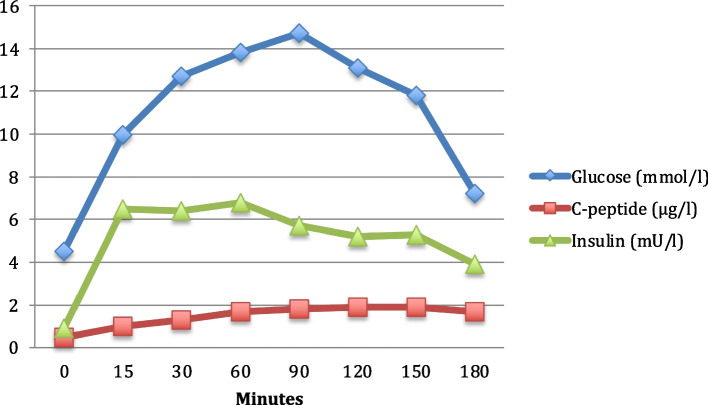
Results of a 1.75 g Glucose/kg body weight oral glucose tolerance test (180 min, 8 measurement points) in our patient with Schwachman-Diamond syndrome at the age of 4.9 years. Blue diamonds indicate venous plasma glucose (mmol/l), green triangles insulin (mU/l), and red squares represent c-peptide (µg/l) levels before (0), and 15, 30, 60, 90, 120, 150, and 180 min after oral glucose load, respectively

Beginning at 1.8 years of age, self-monitored blood glucose levels were significantly elevated, and HbA1c increased to 6.8%, indicating manifest diabetes mellitus. At the same time, spontaneous hypoglycemic episodes persisted. Table 2 details the progression of diabetes with a gradual increase in the autoimmune process and the key characteristics of this case of SDS-associated diabetes mellitus are summarized in Table 3. The mild progression of diabetes, combined with feeding difficulties and, most importantly, the occurrence of spontaneous, ultimately unexplained hypoglycemic episodes, led to the decision to postpone initiation of insulin therapy at this stage. Tapering of the hydrocortisone dose and repeated modifications of the tube feeding regimen led to a relative improvement of hyperglycemic as well as low-normal to hypoglycemic episodes at this stage. After these interventions, HbA1c dropped to 6.2% at two years of age, but glycemia remained unstable. Subsequently, up to the age of 4 years, HbA1c gradually increased again (Table 2), and continuous glucose monitoring (CGMS) was initiated. CGMS data revealed a slowly deteriorating glucose homeostasis with prolonged periods of hyperglycemia after tube feeding (Fig. 1). A steady and unfavorable decline in time in range during her fifth year of life (time in range 48%, time above range 47%, time below range 5%, glucose variability 43%, cumulative CGMS data over three months from 4.6 to 4.9 years of age) led to a diagnostic reassessment and reevaluation of therapeutic options at 4.9 years of age. A standard-dose OGTT with extended measurements of glucose, insulin, and c-peptide was performed, revealing rigid insulin and c-peptide secretion consistent with marked beta-cell dysfunction and severely reduced beta-cell capacity (Fig. 2).

**Table 2:** HbA_1_c and autoimmunity progression in the reported patient with Shwachman-Diamond syndrome (NR, normal range)

**Age **(years)	1.8	2.1	2.4	3	4
**HbA**_**1**_**c** (%)	6.8	-	6.2	6.1	6.5
**GAD antibodies** (NR < 5 U/ml)	25.4	54.9	81.6	-	-
**IA2 antibodies** (NR < 15 U/ml)	< 0.3	< 0.3	< 0.3	-	-
**Insulin antibodies** (NR < 10 U/ml)	0.5	0.9	1.8	-	-

**Table 3:** Clinical and laboratory characteristics of Shwachman-Diamond syndrome-associated diabetes in the here reported patient

**Key features of diabetes mellitus in the reported patient with SDS**
age at diabetes diagnosis < 2 years
prodromal phase with spontaneous episodes of hypoglycemia before the diagnosis of diabetes mellitus
impaired beta-cell responsiveness to oral glucose load with rigid pattern of insulin secretion without clear first and second phases
slowly progressive dysglycemia with high glucose variability and persisting episodes of spontaneous hypoglycemia without antidiabetic treatment
slowly increasing levels of GAD-autoantibodies
no response to treatment with sulfonylurea
maintenance of adequate glycemic control (TIR > 70%), and significant reduction of glucose variability (< 35%) and of hypoglycemic episodes ≥ 12 months using high-fiber tube feed formula diet only

**Fig. 3:**
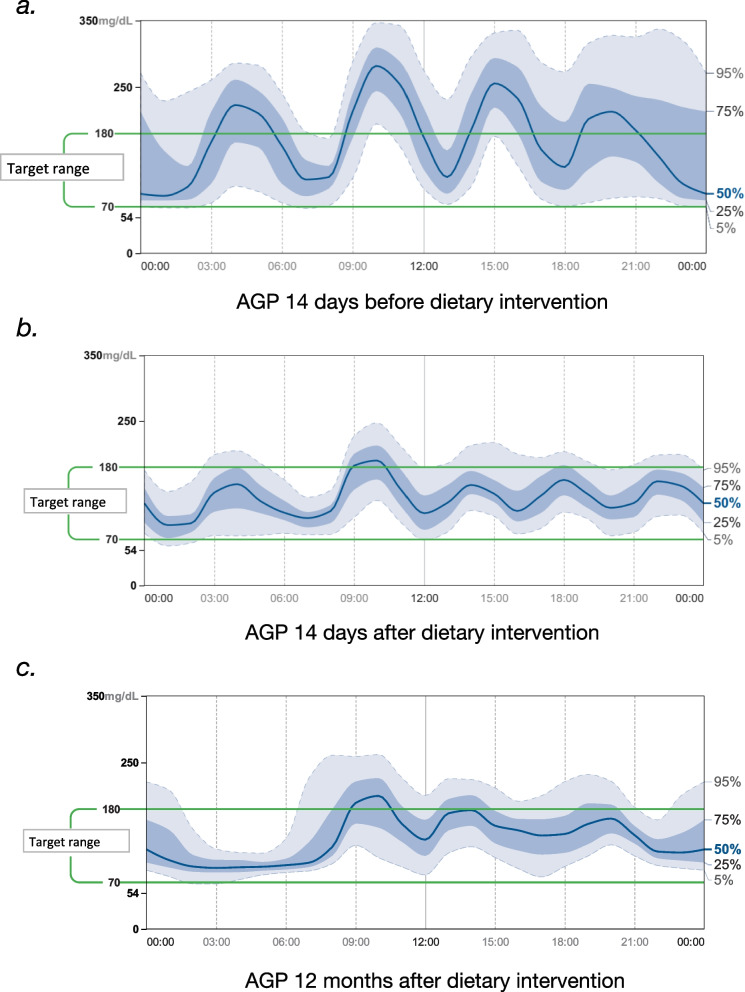
Comparison of 2-week glycemic profiles (AGP) of our patient with Schwachman-Diamond syndrome before (**a**), directly after (**b**), and 12 months (**c**) after sustained dietary intervention (high-fibre formula) without any other antidiabetic treatment

The clinical constellation of manifest diabetes mellitus in a child with marked neurocognitive retardation, failure to thrive, and unexplained spontaneous hypoglycemic episodes, in addition to exocrine pancreatic insufficiency and complete dependency on gastric tube feeding, presents a major therapeutic challenge. After consideration of standard (e.g., insulin) and experimental (e.g., GLP-1 analogues) treatment options, a short inpatient trial of glibenclamide was initiated. Sulfonylurea administration had no beneficial effect on the glycemic profile (7-day CGMS data: time in range 49%, glucose variability 47.2%, mean glucose 178 mg/dl; maximum administered glibenclamide dose 0.2 mg/kg/d) and was subsequently discontinued. As sulfonylurea treatment proved unsuccessful, a new formula (Nutrison advance Diason, Nutricia) with a specifically low glycemic index was introduced prior to transition to insulin pump therapy. Figure 3 illustrates the glycemic control before (Fig. 3a) and after the introduction of the high-fiber formula diet (Fig. 3b), showing improved glycemic variability with significantly fewer hyperglycemic episodes and almost no hypoglycemic episodes. The time in range before and after the dietary intervention for the two-week period was 62% and 81%, respectively. This provided a basis for delaying the initiation of insulin therapy, which is associated with a greater risk of hypoglycemia. Favorable glycemic control has been maintained over a now twelve-month follow-up period of continued tube feeding with a high-fiber formula. Recent 14-day ambulatory glucose profiles indicate time in range (sensor glucose 70 to 180 mg/dl) of 78%, 19% of readings in the 181 to 250 mg/dl range, and only 2% of readings above 250 mg/dl. Reassuringly, only 1% of readings were below 70 mg/dl and no hypoglycemic episodes (sensor glucose < 54 mg/dl) occurred (Fig. 3b). Stabilization of glucose variability was also sustained, with all analyses of the coefficient of variation over the past 12 months remaining below 35%.

### Growth retardation

**Fig. 4:**
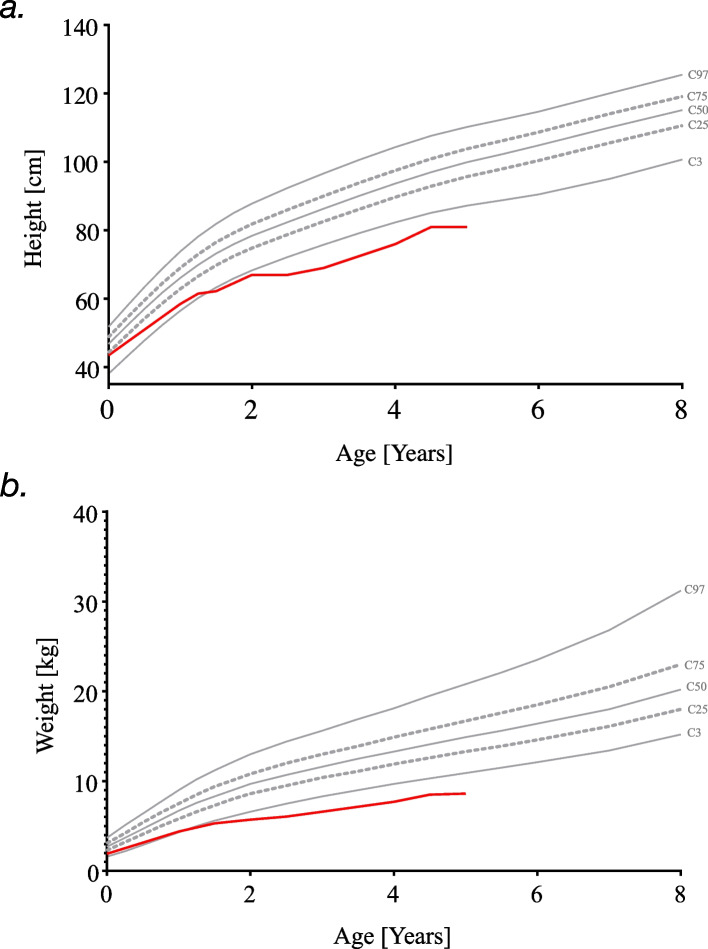
Height **a** and weight **b** development of the girl with SDS based on specific SDS charts from the Italian cohort [30]

Basic laboratory screening for growth hormone deficiency revealed IGF-1 and IGFBP-3 levels in the lower normal range (IGF-1 48 ng/ml, reference range: 22–145; IGFBP-3 1598 ng/ml, reference range: 1388–4151). At 4.9 years of age, weight and height were 5.72 kg and 67 cm, respectively (< 3rd percentile for age and sex, respectively). Figure 4 shows the girl's growth charts, which indicate severe retardation in height and weight. Since growth hormone deficiency could at least contribute to the recurrent hypoglycemia, two growth hormone stimulation tests with arginine infusion were performed. These tests showed a maximum peak secretion of hGH of 10.8 μg/l, which excluded growth hormone deficiency. No other endocrinopathies were detected.

## Discussion and conclusions

We report an early, mild, and prolonged manifestation of type 1 diabetes in a girl with a severe clinical, and specifically hematological phenotype of Shwachman-Diamond syndrome, who underwent bone marrow transplantation at 3 months of age. Of note, our patient is one of only two patients described in the literature who underwent HSCT at such a young age [31].

Diabetes mellitus was diagnosed at 1.8 years of age and followed a mild course with initially tolerable postprandial hyperglycemia and relatively preserved beta-cell function for almost 3 years. Interestingly, even before the diagnosis of diabetes, glycemia was unstable with spontaneous hypoglycemic episodes. Although the diagnostic workup of hypoglycemia was challenging, in retrospect the following can be summarized: Dynamic stimulation testing and repeated basal laboratory screening ruled out severe growth hormone deficiency and adrenal insufficiency as potential endocrine causes. Prolonged 3-h OGTT showed no evidence of excessive insulin secretion after glucose loading with subsequent hypoglycemia in the sense of a "dumping syndrome". However, the boli of tube feeding administered in a comparatively short time may have induced a dumping phenomenon in the patient because, interestingly, the blood glucose increases after feeding were greater than those after administering the dextrose solution. In addition, glycemic variability and especially the frequency of hypoglycemia improved significantly after switching to the high-fiber tube feed.

Baseline screening for inborn errors of metabolism was unremarkable, but very rare metabolic disorders could not be ruled out with absolute certainty because no diagnostic blood sample could be successfully obtained during hypoglycemia.

In the present case, the development of SDS-associated diabetes resembles cystic fibrosis-related diabetes (CFRD) [32] combined with spontaneous recurrent hypoglycemic episodes rather than typical type 1 diabetes: The manifestation of diabetes is mild, prolonged, and does not require intensive insulin treatment. In CFRD, impairment of intracellular and membrane polarization regulation alters beta-cell function and leads to a decrease in first-phase insulin release [33]. However, the impairment of beta-cell function is typically less severe, at least in the early phase of CFRD, than in the case of SDS-associated diabetes described here. Other pathways for the development of dysglycemia have been described in other exocrine pancreatic diseases due to pancreatic inflammation or absolute loss of pancreatic alpha- and beta-cells, hypothetically comparable to SDS [34]. It should be noted that the question arises whether the observed antibody positivity might be an unrelated epiphenomenon due to immunoglobulin substitution or due to an autoimmune process targeting beta-cells as in type 1 diabetes. In our opinion, the gradual but steady increase of at least one marker of beta-cell autoimmunity over time is most likely compatible with the diagnosis of autoimmune type 1 diabetes mellitus in our patient. However, it has to be considered that in the present case the patient's immune system is completely donor derived. Therefore, alloreactive antibodies could originate from alloreactive plasma cells, since the patient has no functional B cells (no class-switched B cells), and furthermore, one could postulate that alloreactive T cells (from the donor) have the potential capacity to destroy beta-cells. Although the underlying immunologic mechanisms in the present case are still unclear, the clinical course to date and the laboratory parameters obtained would be consistent with our hypothesis that SDS-associated type 1 diabetes in early childhood is characterized by a milder course with a prolonged period of dysglycemia and relatively preserved beta-cell capacity.

Based on previously described cases in the literature, we hypothesize that there are two groups of diabetic SDS patients depending on the age of diabetes manifestation: early manifestation during infancy and early childhood, characterized by a milder course and slow progression, and late manifestation during adolescence or even adulthood, requiring intensive insulin therapy from diagnosis. We suggest careful monitoring and investigation of the glycemic pattern in other less severe cases of SDS to identify the presence of a mild and long presymptomatic period of dysglycemia. This will allow clinicians to gain appropriate knowledge and new insights into glucose metabolism and the early onset of diabetes in patients with SDS.

Further study of the age-related patterns of type 1 diabetes development and the possible application of dietary interventions in early-onset SDS-associated diabetes should be priorities for further investigation.

### Learning points

- In younger children with Shwachman-Diamond syndrome, diabetes mellitus seems to be characterized by a milder manifestation and clinical course, probably associated with nontypical symptoms (hypoglycemia),
- Dietary interventions providing low glycemic index feeding should be considered an important adjunct to more intensive treatment strategies (e.g., insulin pump therapy).
- Careful monitoring of glycemic patterns among nonsevere cases of Shwachman-Diamond syndrome could identify the presence of a mild and prolonged course of presymptomatic dysglycemia.

## Data Availability

Data sharing is not applicable to this case report because no datasets were generated or analyzed during the study.

## Abbreviations

SDS: Shwachman-Diamond Syndrome
CFRD: Cystic fibrosis-related diabetes
hGH: Human Growth Hormone
OGTT: Oral glucose tolerance test
HSCT: Haematopoietic stem cell transplantation
GvHD: Graft-versus host disease
IGF-1: Insulin-like peptide-1
GLP-1: Glucagon-like peptide -1
IFG-BP-3: IGF binding protein-3
NR: Normal range
CGMS: Continuous glucose monitoring system
HbA1c: Glycose-binded haemoglobin 1c
IgG: Immunoglobulin G
